# Machine learning–inspired design of a modified spider-shaped MIMO antenna for 5G-advanced wireless communications

**DOI:** 10.1371/journal.pone.0355221

**Published:** 2026-08-06

**Authors:** Ashwini Kumar, Ashish Kumar, Gurprince Singh, Neeraj, I. M. Ibrahim, A. J. A. Al-Gburi

**Affiliations:** 1 Deaprtment of Computer Engineering, Poornima Institute of Engineering and Technology, Jaipur, India; 2 Chitkara University Institute of Engineering and Technology, Chitkara University, Punjab, India; 3 Department of Electronics and Communication Engineering, Thapar Institute of Engineering and Technology, Patiala, Punjab, India; 4 Department of Computer Science Engineering, Galgotias University, Greater Noida, Uttar Pradesh, India; 5 Center for Telecommunication Research & Innovation (CeTRI), Fakulti Teknologi Dan Kejuruteraan Elektronik Dan Komputer (FTKEK), Jalan Hang Tuah Jaya, Durian Tunggal, Melaka, Universiti Teknikal Malaysia Melaka (UTeM), Malacca, Malaysia; 6 Strategic Research Institute (SRI), Asia Pacific University, Jalan Teknologi 5, Taman Teknologi Malaysia, Kuala Lumpur, Malaysia; Guangdong University of Petrochemical Technology, CHINA

## Abstract

A modified Spider Shaped Four Element Multi Input Multi Output Antenna (SSFEMIMOA) is suggested for the 5G-advanced, and sub-6 GHz advanced wireless communications. The proposed SSFEMIMOA exhibits a low-profile and compact configuration with an overall size of 0.36λ² (52 × 52 × 1.57 mm³), achieved through the optimal placement of the constituent antenna elements. The top layer of the unit-cell antenna is designed in a modified spider-shaped geometry incorporating an additional oval-shaped structure. The modified partial ground structure alters the current distribution and electromagnetic coupling within the antenna, thereby improving impedance matching and broadening the operating bandwidth. The diversity parameters of the SSFEMIMOA have been attained by placing the elements in orthogonal position to each other. The anticipated design attains the BW ≈ 5.5 GHz (3.5–9.05) with maximum gain of 6.1 dB and isolation between −20 to −40 dB while satisfying the MIMO diversity parameters in terms of Envelope Correlation Coefficient (ECC), Directive Gain (DG), Channel Capacity Loss (CCL) and Total Active Reflection Coefficient (TARC). To enhance the design optimization process, multiple Machine Learning (ML) algorithms were employed and systematically evaluated. Based on the obtained performance metrics, Gaussian Process Regression (GPR) exhibited the best predictive capability, yielding the lowest prediction error and highest accuracy compared to the other investigated algorithms. Finally, the proposed SSFEMIMOA was fabricated and validated through measurements of S-parameters, transmission coefficients, radiation characteristics, and other relevant performance parameters. The experimental results exhibit excellent impedance matching and radiation performance, making the antenna a promising candidate for emerging 5G/6G advanced wireless communication systems.

## 1. Introduction

In modern wireless communications, higher channel capacity, multiband operative antennas, high data rate, system stability and reliability are some of the key features [1]. Conventional Single-Input Single-Output (SISO) systems are inadequate for addressing the challenges posed by the rapidly increasing number of users and network congestion in multipath fading environments. Consequently, their ability to provide high capacity, reliable communication, and improved spectral efficiency is limited [2]. Therefore, MIMO systems have been popular in the recent times to enhance the overall reliability, data rate and other above-mentioned features [3]. However, the close proximity of the antenna elements causes the issue of mutual coupling which in turns effects the performance parameters. Therefore, various techniques like decoupling networks, defected ground structure (DGS), slotted designs, neutralizing lines etc. have been used by the researchers to overcome this issue [4]. Moreover, wide band antennas are also popular among researchers and antenna designers due to lower power consumption, which supports various applications including medical imaging and radar systems [5]. This article primarily focuses on the target 5G and 6G frequency bands, which enable advanced applications such as digital twin technology, seamless data connectivity and control, and high-quality video services in modern wireless communication systems [6]. Furthermore, 6G is expected to build upon the fundamental capabilities of 5G by offering significant enhancements in energy efficiency, positioning accuracy, sensing performance, and overall network reliability. The frequency ranges under 10 GHz that encompass the particular range of frequencies for the desired use cases are included in several papers. In such attempts, the authors in [7] presents the design and ML-based optimization of a miniaturized double port CPW-fed ultra-wideband (UWB) MIMO antenna for next-generation wireless communication. The antenna achieves an 8.7 GHz BW (2.78–11.48 GHz), covering multiple 5G NR, Wi-Fi and vehicular communication bands, with strong isolation and stable performance. Various machine learning (ML) algorithms were employed for design optimization, among which the Decision Tree model demonstrated the best performance, achieving an accuracy of up to 99.92%. In another similar work [8], the authors present a compact, low-profile, d-shaped super UWB (2.5–50 GHz) 4-port MIMO antenna for satellite, 5G, and IoT applications. It achieves over 320% impedance BW with high isolation (>25 dB), low ECC (<0.013), and stable peak gain. A compact four-element UWB antenna with an optimized substrate and defected ground plane achieves inter-element isolation better than −16 dB; however, the modified substrate introduces fabrication challenges. [6]. In another article, a four cell MIMO antenna design for mm wave use cases is discussed with broad band and high gain features. However, the realized DGS is multifaceted in nature [9]. A low frequency four input antenna is designed with outstanding impedance matching and separation for the required use cases [10]. Flexible conductive material and a partial ground plane are used in the design of a flexible wideband MIMO antenna [4]. A spider structured fractal antenna is presented with various performance parameters along with the specific absorption rate (SAR) without any discussion of CCL [11]. Further, the authors of this paper [12] present a 1 × 2 DGS-based fractal antenna intended for sub-6 GHz 5G automotive use cases. It covers key frequency bands (0.7, 2.6, 3.1, and 3.5 GHz), achieves high gain up to 12.9 dB, excellent isolation, and low ECC, making it appropriate for incorporation into automobile body components. In another similar design, the authors propose a compact dual band four element antenna with double circular polarization for WLAN applications. The design uses four E-shaped monopole elements with connected ground planes and an I-shaped strip to ensure voltage uniformity and improve isolation [13]. In [14], a wideband four port antenna array was presented for 5G NR band. The antenna has an overall footprint of 30 × 40 × 1.6 mm^3^ and it operates from 3.2–5.85 GHz. A flexible interconnected four port MIMO antenna with low profile of 0.612λ^2^ is presented in [15]. It covers the Sub-6 GHz, 5G and X-band use cases with a maximum gain ≈ 4 dB and isolation ≈ 20 dB. In [16], a quad-port antenna array was proposed that covers 3.4–3.6 GHz and 4.8–5 GHz for 5G smartphone use cases with an isolation ≈ 16.5 dB. An L-shaped strip, a parasitic rectangular strip, and a modified Z-shaped strip make up the single element. In [17], a four-element self-isolated flexible MIMO antenna with dimensions of 160 × 40 × 0.5 mm³ was proposed, achieving an inter-element isolation greater than 26 dB and a peak gain ≈ 5.3 dB. The development of compact antennas with low cross-polarization discrimination and adequate gain remains a relatively unexplored research area with significant potential for C-band and emerging 6G communication applications. The performance of the antenna design is dependent upon the certain dimensional parameters, which needs to be optimized. One method is to use the trial and error, which consumes a lot of time and resources. Therefore, ML is highly accurate and reliable method to optimize the same. It finds applications in various domains including image processing, biomedical applications, automated translation etc. In this paper, various algorithms have been utilized to optimize the dimensional parameters to achieve the required performance specifications.

A detailed analysis of existing UWB MIMO antenna designs has been discussed to identify key limitations in the current state-of-the-art, such as limited impedance bandwidth, insufficient isolation (typically around −20 dB), larger physical dimensions, and relatively higher ECC. Although some designs achieve wide bandwidth, they often do so at the expense of compactness or inter-element isolation, whereas others provide excellent isolation but fail to deliver wideband performance. Based on the above analysis, the identified research gap is the absence of a compact four-port MIMO antenna capable of simultaneously achieving wide impedance bandwidth, high inter-element isolation (>20 dB), low ECC < 0.05, and stable gain, without increasing the overall antenna size.

This article discusses the design process of miniaturized wideband modified SSFEMIMOA for 5G-advanced wireless communications. The anticipated structure has an overall size of 52 × 52 × 1.6 mm^3^. The antenna elements have been placed in orthogonal position with curved partial ground plane and fed with 50 Ω conventional microstrip feed line. The antenna is analyzed by high frequency structure simulator (HFSS) software. The major contribution of the work is as follow:

1. A SSFEMIMOA is developed that achieves a wide impedance BW of 5.5 GHz (3.5–9.05) without the use of additional decoupling structures, while maintaining the degree of attenuation level (Isolation) better than 20 dB across the operating band.
2. Radiation patterns show good agreement in both XZ- and YZ-planes at representative frequencies, demonstrating stable radiation characteristics over wideband operation.
3. The proposed SSFEMIMOA exhibits robust wideband MIMO performance, with near-zero ECC, high DG, low CCL, and stable TARC across the entire frequency range.
4. An exceptionally wide fractional BW ≈ 88% and a maximum realized gain of about 6 dB are achieved, supporting a range of wideband wireless communication applications.

## 2. Antenna design considerations

**Fig 1:**
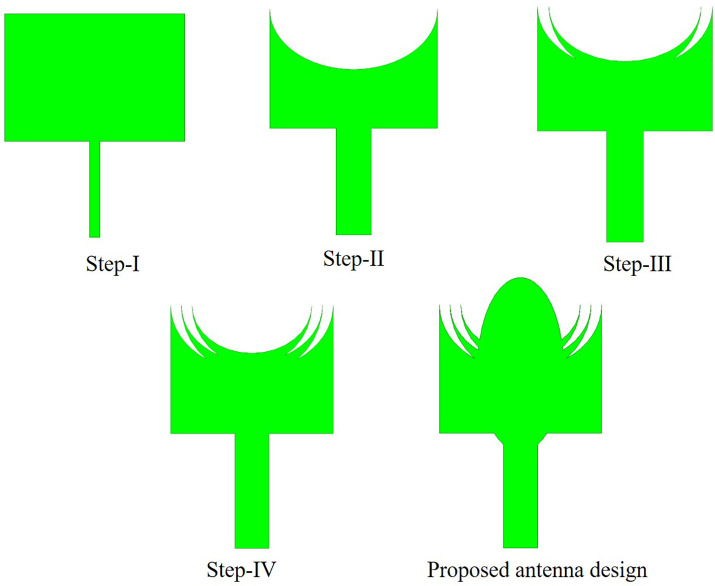
Evolution of the proposed antenna element.

The anticipated design starts with the rectangular patch that has a footprint of 25.2 × 27.8 × 1.6 mm^3^. The substrate used is RT Duroid 5880^TM^ material is used as substrate which has tan δ = 0.0009, ε_r_ = 2.2, and h = 1.57 mm. The initial antenna dimensions were calculated using the standard design equations reported in [18]. The conventional design resonates at 7.3 GHz with impedance BW ≈ 290 MHz (7.16–7.45 GHz). The evolution steps to reach the anticipated design are shown in Fig 1, which is not only conceptual but are guided by quantitative electromagnetic performance improvements at each stage of design. In Step-I, the basic rectangular radiator provides a fundamental resonance with limited bandwidth. In Step-II, the introduction of a semi-circular cut modifies the current path length, resulting in a measurable shift in resonant frequency and improved impedance matching. In Steps III and IV, the incorporation of multiple curved slots further perturbs the surface current distribution, generating additional resonant modes that significantly enhance the impedance BW. Finally, the proposed geometry integrates these features to achieve optimized performance, including wider impedance BW, improved gain, and enhanced isolation. Thus, each geometrical modification is quantitatively justified through its impact on key antenna parameters such as resonance, bandwidth, and return loss. A partial ground plane was employed throughout all design steps to improve the impedance bandwidth. However, the desired bandwidth was still not achieved. Consequently, the partial ground plane was further optimized by chamfering its corners, and the geometric parameters along with the corresponding parametric results from all four design steps were incorporated into the machine learning (ML) training dataset for design optimization. This trained dataset is used not only to obtain best parameters, but it gave the best possible number of semi-circular cut to achieve wideband with best possible reflection coefficient (S_11_). The best-performing model (GPR) predicted the optimized parameters as: L1 = 12 mm, L2 = 9.68 mm, L6 = 11.4 mm, W1 = 15.2 mm, W2 = 3.2, GL = 9, and number of semi-circular slots ≈ 2. A detailed analysis of the machine learning (ML) approach, including the algorithms employed and their corresponding error metrics, is presented in Section 6 of this manuscript.

**Fig 2:**
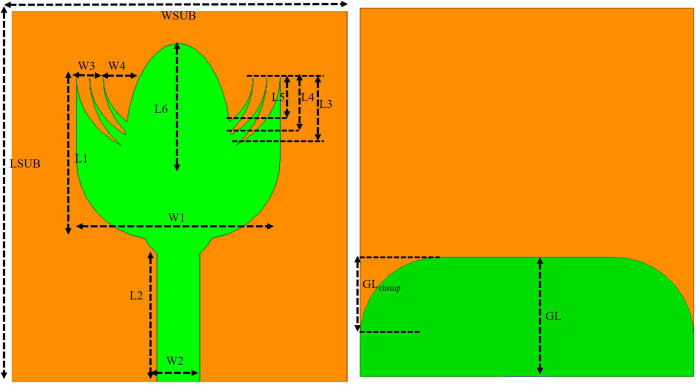
Development of the anticipated antenna design [LSUB = 27.8, WSUB = 25.2, L1 = 12, L2 = 9.68, L3 = 4.7, L4 = 3.7, L5 = 2.7, L6 = 11.4, W1 = 15.2, W2 = 3.2 mm, W3 = 2, W4 = 2.6, GL = 9, GL_champ_ = 6], All dimensions are in mm.

**Fig 3:**
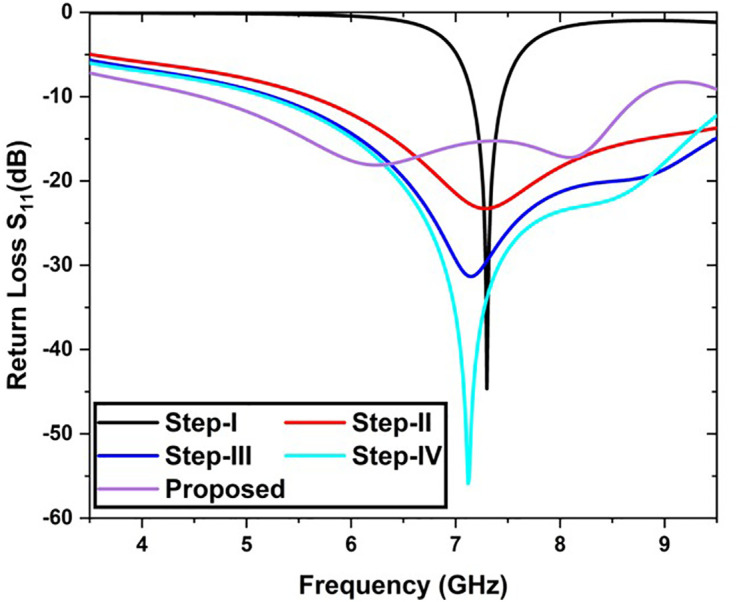
S_11_ of the propose design at different stages.

The dimensional pictorial view of the antenna element is shown in Fig 2. Further, the S_11_ and impedance BW performance are shown in Fig 3, which depicts that the conventional rectangular patch, i.e., step-I resonates at 7.3 GHz with excellent impedance matching and narrow −10 dB impedance BW ≈ 290 MHz (7.16–7. 45 GHz).

**Fig 4:**
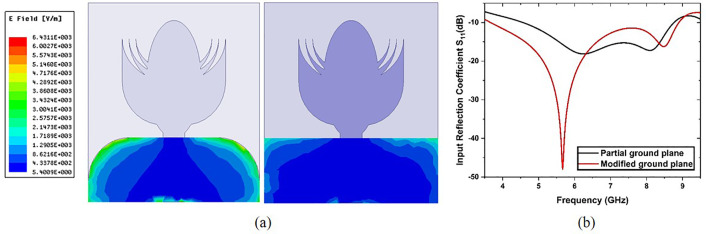
Impact of Modified Ground plane on S_11_ of the propose design (a) Current distribution (b) S_11_ v/s frequency.

**Fig 5:**
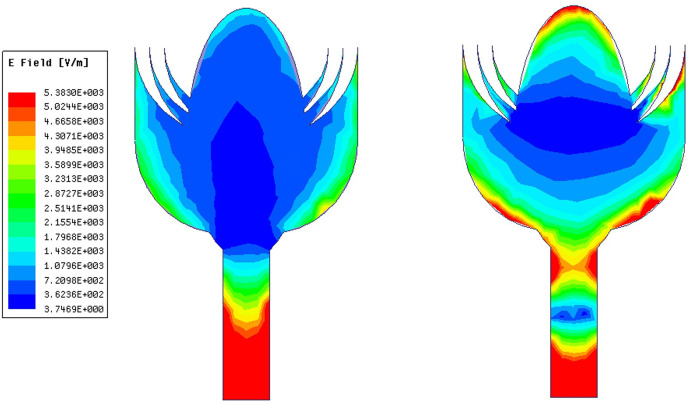
E-field distribution (a) 5.67 GHz (b) 8.5 GHz.

Further, step-II achieves the same resonating frequency with improved BW ≈ 3.83 GHz (5.67–9.5 GHz). Similarly, further modifications in the design have been performed in terms of step-III and step-IV which also resonates near 7.1 GHz and impedance BW ≈ 4.25 GHz (5.25–9.5 GHz). However, final proposed design also covers the same frequency band with similar BW values but with poor impedance matching in entire operating band as shown in Fig 3. Therefore, partial ground is modified into curved corners as illustrated in Fig 2. The performance comparison is depicted in Fig 4, in which the performance is exceptionally improved in terms of S_11_ values (S_11_ ≈ −48 dB) and impedance BW ≈ 5.67 GHz (3.66–8.9 GHz). The improved performance of the proposed antenna with the modified ground plane is attributed to the redistribution of surface currents and electric fields around the radiator-ground interface. The ground-plane modification increases the effective current path length and introduces additional capacitive-inductive loading, which enhances the electromagnetic coupling between the radiator and ground plane. As shown in Fig 4(a), a stronger and more uniform electric field distribution is observed for the modified ground plane compared to the conventional partial ground plane. This field redistribution improves impedance matching, reduces the reflection coefficient, and results in a wider operating bandwidth. Hence, the modified ground plane serves as an effective impedance-tuning structure that enhances the overall radiation performance of the antenna. The far field features of the unit cell are expresses in term of 2D radiation patterns. The anticipated antenna design exhibits the gain of 3.4 and 4.6 dB at 5.67 and 8.5 GHz respectively. The shape of the pattern can be examined from the current distribution at the respective resonating frequencies in Fig 5.

**Fig 6:**
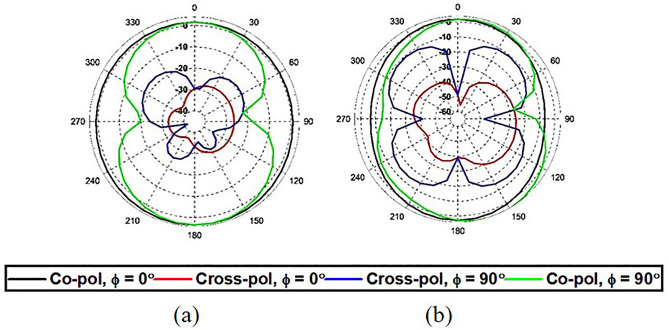
2D-radiation patterns (a) 5.67 GHz (b) 8.5 GHz.

**Fig 7:**
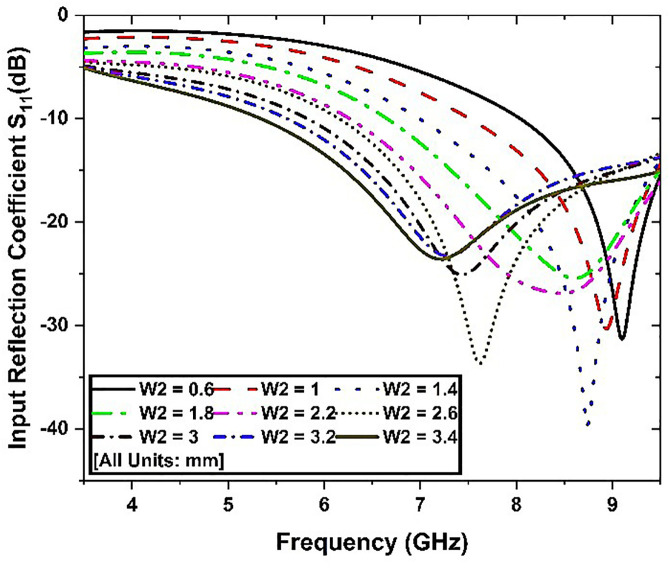
Feed Width (W2) variation effect on S_11_.

**Fig 8:**
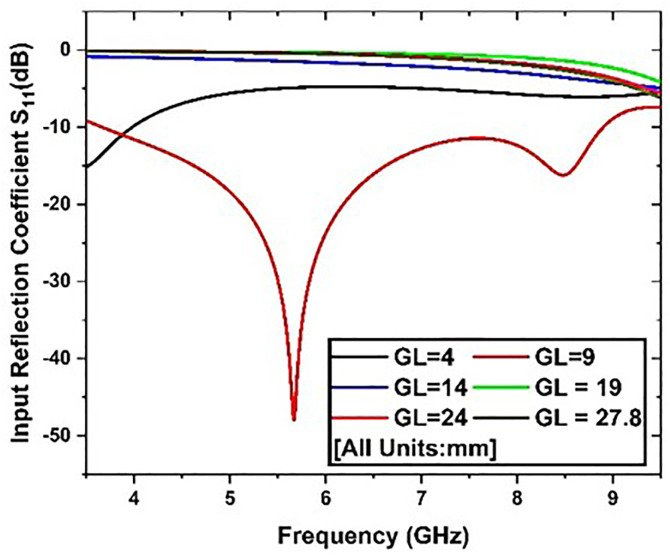
Ground length (GL) Effect on S_11_.

For instance, at 5.67 GHz the current density is minimized at the central region while strong current concentration occurs along the edges of the radiating structure. This distribution indicates that the effective radiation is primarily governed by edge currents, which act as radiating slots, producing a nearly uniform radiation in the azimuth plane and thus resulting in an omnidirectional pattern. Furthermore, the symmetric current distribution ensures balanced field cancellation in the broadside direction and constructive radiation along the periphery. At 8.5 GHz, the emergence of additional current paths and non-uniform field distribution confirms the excitation of higher-order modes, which slightly modifies the radiation characteristics. These observations are now supported with fundamental electromagnetic principles, including current continuity, boundary conditions, and modal behaviour of patch-like structures, to provide a more quantitative and physics-based interpretation of the antenna operation. Fig 6 shows the 2D radiation patterns at 5.67 and 8.5 GHz. The impedance matching and the wide BW depends upon the certain dimensional parameters (W2 and GL). Although Figs 7 and 8 present the variation of S_11_ with respect to W2 and GL, the observed trends are fundamentally governed by changes in impedance matching and current distribution within the antenna structure. Specifically, the feed width (W2) directly controls the characteristic impedance of the microstrip feed line; increasing W2 improves the impedance matching between the feed and the radiating element, which results in a deeper S_11_ and enhanced bandwidth. Similarly, the ground plane length (GL) plays a critical role in determining the effective current path and the formation of fringing fields. Variations in GL alter the distribution of surface currents and the coupling between the radiator and ground plane, thereby shifting the resonant frequencies and affecting bandwidth. An optimal GL ensures proper current formation and stable radiation, while non-optimal values lead to detuning and reduced performance.

## 3. MIMO antenna design

**Fig 9:**
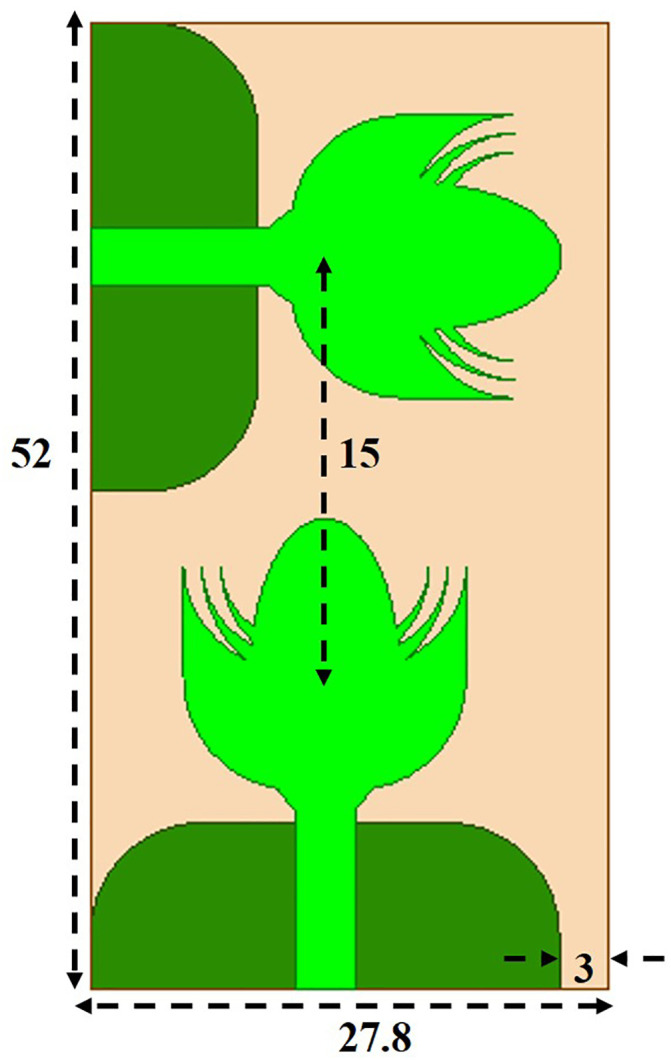
SSTEMIMOA MIMO structure (all dimensions are in mm).

**Fig 10:**
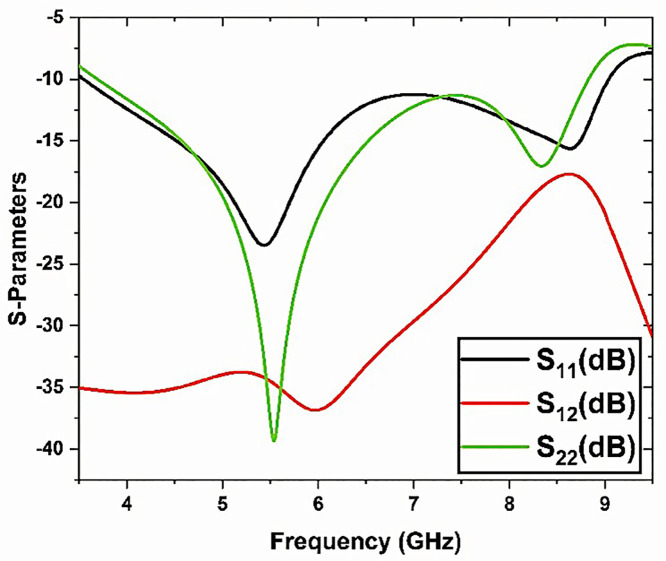
S-Parameter of SSTEMIMOA.

**Fig 11:**
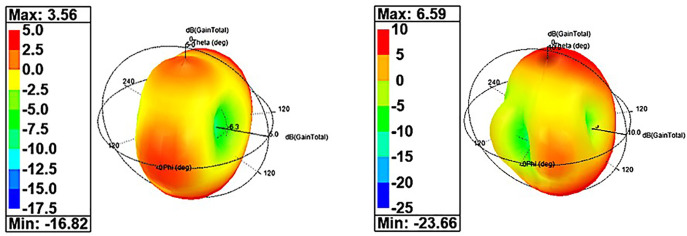
3D-polar plot of SSTEMIMOA at 5.67 GHz and at 8.49 GHz.

This section examines the performance of a 1 × 2 and 2 × 2 MIMO antenna structure. The geometry of the anticipated Spider Shaped Two Element Multi Input and Multi Output Antenna (SSTEMIMOA) design is illustrated in Fig 9. Fig 10, shows the S-parameter of the SSTEMIMOA structure which depicts the wide band operating BW ≈ 5.5 GHz (3.5–9.05 GHz) for both the ports and isolation between the ports is well below the −18 dB in whole band. Moreover, the 3D gain polar plot is also illustrate in Fig 11, which mention the gain 3.5 and 6.6 dB at 5.67 and 8.5 GHz respectively.

**Fig 12:**
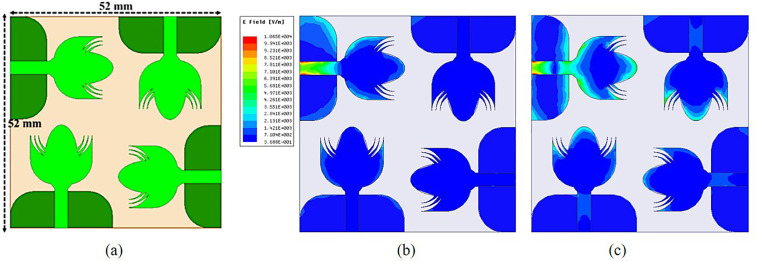
Proposed SSFEMIMOA (a) Structure (b) Current distribution at 5.67 GHz and (c) Current distribution at 8.49 GHz.

**Fig 13:**
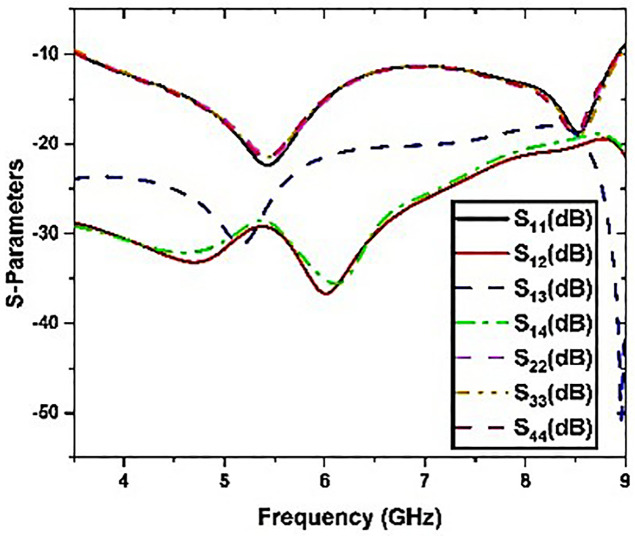
S-Parameter of SSFEMIMOA structure.

**Fig 14:**
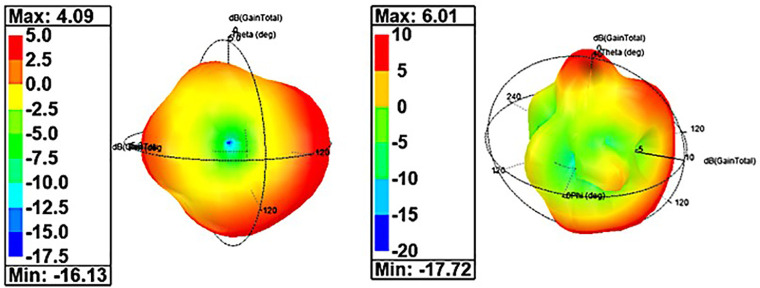
3D-polar plot of SSFEMIMOA structure at 5.67 and 8.49 GHz.

Fig 12(a), shows the structure of SSFEMIMOA antenna structure in which the elements are arranged in orthogonal position. The E-field distributions shown in Fig 12(b) & (c) provide clear insight into the coupling mechanisms between the MIMO elements at different operating frequencies. At 5.67 GHz, the electric field is predominantly concentrated around the feed region and the central radiating patch, while only weak field intensity is observed on the adjacent elements. This distribution indicates minimal mutual coupling and effective inter-element isolation, primarily due to the optimized slot geometry and adequate spatial separation between the antenna elements. In contrast, at 8.49 GHz, the E-field becomes more distributed along the edges and the curved slot regions, where fringing fields and surface wave interactions are more pronounced. However, the presence of the semi-circular cuts and slot perturbations effectively suppresses surface current propagation toward neighbouring elements, thereby reducing coupling paths. The S-parameter performance of the SSFEMIMOA structure, illustrating both reflection and mutual coupling behaviour among the ports is shown in Fig 13. The reflection coefficients (S₁₁, S₂₂, S₃₃, and S₄₄) exhibit multiple resonances, with prominent impedance matching observed around 5.5–6 GHz and near 8.5 GHz, indicating efficient radiation and minimal return loss at these frequencies. The transmission coefficients (S₁₂, S₁₃, and S₁₄) represent the isolation between antenna elements; among these, S₁₄ and S₁₂ generally remain below −20 dB across a significant portion of the band, demonstrating good isolation performance, while S₁₃ shows comparatively higher coupling but still maintains acceptable levels (≈ −15 to −20 dB). The variation in coupling levels across frequencies indicates the presence of multiple resonant modes and interaction paths between elements. Overall, the results demonstrate that the proposed antenna achieves wideband operation with excellent impedance matching and effective inter-element isolation, making it well suited for MIMO applications that require low mutual coupling, high isolation, and stable multi-port performance. The 3D gain plot is also shown in Fig 14 which again depicts the gain of 4.09 and 6.01 dB at 5.67 and 8.5 GHz respectively.

## 4. Fabrication and measurements results

**Fig 15:**
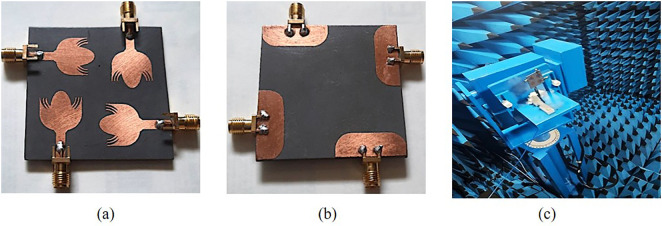
Fabrication (a) top layer (b) Bottom Laye of SSFEMIMOA, and (c) Test Antenna in anechoic chamber.

**Fig 16:**
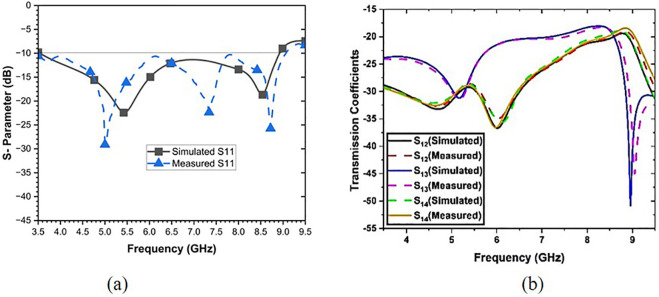
S-Parameter Measured results of SSFEMIMOA structure.

**Fig 17:**
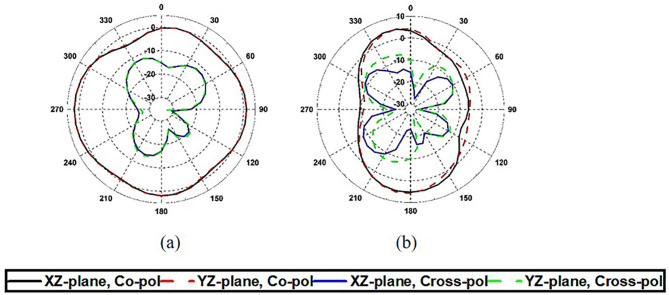
Simulated and Measured Radiation Patterns (a) 5.67 GHz (b) 8.49 GHz.

The fabricated sample of the SSFEMIMOA is revealed in Fig 15(a) and (b) with measurement environment is shown in Fig 15(c). The metallization was carried out using a copper layer of 35 μm thickness, and the footprint of the prototype antenna match those provided in Fig 12. The simulated and measured resonance characteristics are shown in Fig 16(a) and (b), From the graph, both simulated and measured S_11_ responses confirm the wideband operation of the proposed antenna, covering band of 3.5–9.2 GHz. Specifically, at around 3.5–4.5 GHz, S_11_ remains close to −10 dB, indicating the start of the operating band. A significant resonance occurs near 5.2 GHz, where the simulated S_11_ reaches approximately −22 dB and the measured value drops further to ≈ −30 dB, indicating excellent matching. This resonance is primarily associated with the fundamental current path established along the radiator and the ground plane. Around 6.5–7 GHz, both curves stabilize near –12 to –14 dB, still within acceptable limits. Another dip appears near 7.5 GHz, where measured S_11_ drops to ≈ –22 dB, while the simulated value remains around –12 dB. This behavior can be attributed to higher-order current modes and additional capacitive-inductive coupling introduced by the modified ground structure. At higher frequencies, around 8.5–9 GHz, a strong resonance is again observed, with measured S_11_ reaching ≈ –25 dB and simulated ≈ –18 dB. This resonance is associated with higher-order surface current distributions and enhanced electromagnetic coupling between the radiator and the ground plane. Finally, near 9.5 GHz, both curves rise toward –8 to –10 dB, marking the upper band edge. the antenna demonstrates a wide impedance bandwidth from approximately 3.5 GHz to 9.2 GHz. Although the overall agreement between simulation and measurement is good, slight discrepancies in resonance frequency and impedance matching are observed due to various attributes like (a) small deviations in the dimensions of the radiator, feed line, and modified ground plane during fabrication can alter the effective current paths, resulting in resonance shifts, (b) The actual dielectric constant and loss tangent of the substrate may differ slightly from the nominal values used in simulation, particularly over a wide frequency range, (c) The influence of the SMA connector, soldering imperfections, and feed transitions are difficult to model accurately and can introduce additional inductive or capacitive effects, (d) Surface roughness of the copper layer and manufacturing imperfections introduce additional losses that are generally not fully accounted in the electromagnetic model. Further, 2D radiation patterns are depicted in Fig 17, which shows the co-polarization and cross polarization components. It can be depicted that for 5.67 GHz, the radiation pattern is nearly omni-directional and it is directional in case of 8.49 GHz. At both the frequencies, the cross-polarization discrimination is also less than −10 dB.

**Fig 18:**
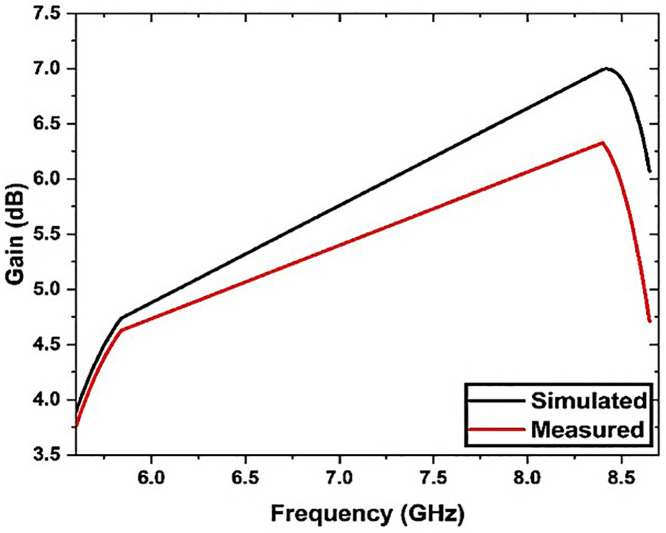
Simulated and Measured Gain.

The simulated and measured gain graph is revealed in Fig 18. At the lower range of frequencies near 5.8 GHz, the gain starts around 3.8 dB for both cases, indicating moderate radiation efficiency. As frequency increases toward 6 GHz, the gain rises sharply to about 5.5–6 dB. Beyond this point, the gain continues to increase almost linearly, reaching approximately 6.5–7 dB near 7.5 GHz in simulation, while the measured gain remains slightly lower at around 6–6.3 dB, likely due to practical losses and fabrication tolerances.

**Fig 19:**
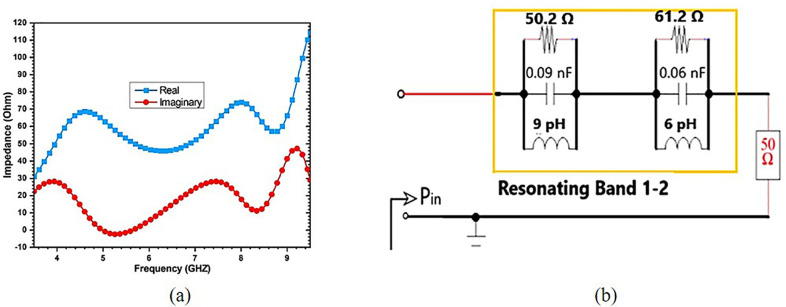
(a) Impedance versus frequency Graph (b) Equivalent Circuit.


Further, the real and imaginary part versus frequency variations has been shown in Fig 19(a), which depicts the real part near the range of 50 Ω. And imaginary part near 0 Ω in the band of interest. Based on the obtained values equivalent circuit has been drawn and shown in Fig 19(b). The equivalent circuit is derived based on the impedance characteristics. Each resonance in the impedance curve corresponds to a parallel RLC tank, where the resistive component represents radiation and loss, the inductance accounts for the current path along the radiating structure, and the capacitance models the fringing fields and coupling effects. The component values are extracted using standard curve-fitting and resonance-based formulations. Where the resonating frequency (*f*) is used to estimate L and C as expressed in equation (1) along with the quality factor is used to determine *R.*

$$\begin{array}{c} f=\frac{\text{1}}{2\pi \sqrt{LC}} \end{array}$$ (1)

## 5. MIMO diversity parameters

The Diversity analysis of the proposed SSFEMIMOA system is required to ensure the reliable performance and enhanced DG especially in multipath signal environments. Also, the adjacent radiating elements for the discussed MIMOA must be perfectly isolated to validate good diversity performance. The diversity performance is evaluated for the proposed SSFEMIMOA design in this section. The evaluated parameters are ECC, DG, and CCL and TARC to validate its utilization for 6G mid-band/upper mid-band Wireless Applications.

(a) ***ECC***

ECC determines the relation between the distinct elements of the MIMOA, in terms of the crucial diversity parameter, ECC, using S-Ps, as detailed in [19] calculated using Eq. (2),(3).


$$\begin{array}{c} {ECC}_{MIMO}=\frac{{\left|S_{\text{1}\text{1}}^{*}S_{\text{1}\text{2}}+S_{\text{2}\text{1}}^{*}S_{\text{2}\text{2}}\right|}^{\text{2}}}{\left(\text{1}-{\left|S_{\text{1}\text{1}}\right|}^{\text{2}}-{\left|S_{\text{2}\text{1}}\right|}^{\text{2}})(\text{1}-{\left|S_{\text{2}\text{2}}\right|}^{\text{2}}-{\left|S_{\text{2}\text{1}}\right|}^{\text{2}}\right)} \end{array}$$ (2)

$$\begin{array}{c} ECC=\frac{\iint_{4\pi }E_{p}\left(\theta ,\phi \right),E_{q}\left(\theta ,\phi \right)d\Omega }{\sqrt{\iint_{4\pi }E_{p}\left(\theta ,\phi \right).E_{p}^{*}\left(\theta ,\phi \right)d\Omega \iint_{4\pi }E_{q}\left(\theta ,\phi \right).E_{q}^{*}\left(\theta ,\phi \right)d\Omega }} \end{array}$$ (3)

**Fig 20:**
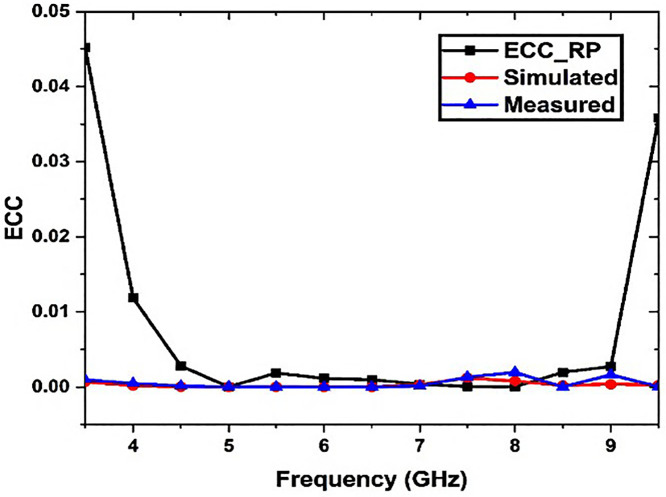
ECC of the proposed design.

The ECC for simulation and measurement results are calculated and plotted as indicated in Fig 20. It is depicted that the designed SSFEMIMOA demonstrates favourable ECC performance in the desirable resonating bands. Thus, the ECC value remains well below the desirable limit of 0.05, confirming uncorrelated operation with robust isolation among various antenna elements, satisfying the desired condition of less than 0.05 for MIMOA applications, and validating consistently low ECC values across all three operational bands.

(b) **DG**

**Fig 21:**
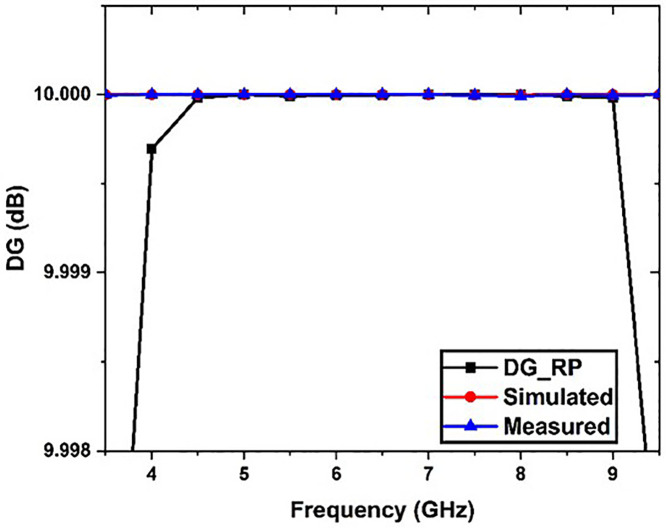
DG of the proposed design.


DG is another crucial parameter to determine the diversity performance of MIMO antenna. It evaluates the Signal-to-Noise (S/N) ratio of the MIMO antenna system in comparison to the single-radiating element and is evaluated using equation 4. The diversity gain has an ideal value of 10. Further, measured results for the DG of designed triple element MIMO antenna are plotted and compared with simulated values in Fig 21. The simulated and measured value in the desired resonant bands are observed to range between 9.999 dB and 10 dB, fulfilling the desired condition for MIMO applications. The value of DG is aligned to the simulated value of 10 dB at the desired bands, including resonant frequencies confirming the significant contribution of the presented MIMO antenna design in overcoming the multipath fading and enhancing the reliability of the wireless link and validating its practical utility for better performance in wireless communication systems.

$$\begin{array}{c} \text{DG}=\text{1}\text{0}\sqrt{\text{1}-{\left|{ECC}_{MIMO}\right|}^{\text{2}}} \end{array}$$ (4)

(c) **CCL**

The channel capacity as specified in Shannon’s theorem, determines the maximum information transmission rate relying majorly on the performance of other diversity parameters increasing linearly with the increase in the number of antenna elements in MIMO system configuration. Thus CCL is the crucial parameter to evaluate the performance of MIMO antenna compared to that of single element and assess the degradation in the system performance calculated using equation (5)–(8) below


$$\begin{array}{c} C\left(loss\right)=-{log}_{\text{2}}det\left(a\right) \end{array}$$ (5)

$$\begin{array}{c} a=\left[\begin{array}{cc} @cc@\sigma_{11} & \sigma_{12}\\ \sigma_{21} & \sigma_{22} \end{array}\right] \end{array}$$ (6)

$$\begin{array}{c} \sigma_{ii}=1-\left({\left|S_{ii}\right|}^{\text{2}}-{\left|S_{ij}\right|}^{\text{2}}\right) \end{array}$$ (7)

$$\begin{array}{c} \sigma_{ij}=-\left(S_{ii}^{*}S_{ij}+S_{ji}S_{jj}^{*}\right) \end{array}$$ (8)

**Fig 22:**
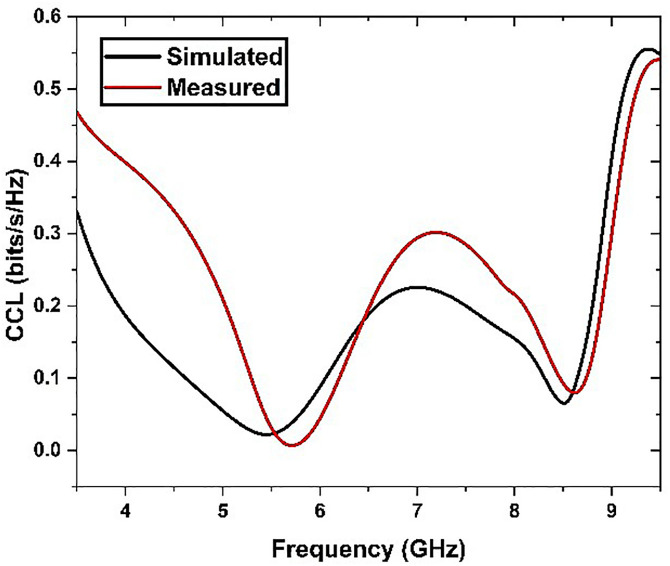
CCL of the proposed design.

The ideal value of CCL must be 0 bits/s/Hz and generally below 0.5 bits/s/Hz to be considered acceptable for high-performance systems. The measured CCL for the designed MIMO antenna compared to its simulated value as shown in Fig 22 are below 0.05 b/s/Hz and 0.0025 b/s/Hz, respectively satisfying the requirement of maintaining CCL below 0.5 bits/s/Hz across the desired resonating bands. Additionally, the designed antenna exhibits CCL = 0.0 bits/s/Hz at resonant frequency of 5.5 GHz, justifying a state of maximum spectral efficiency, signifying high port-to-port isolation, ensuring the system’s high data throughput potential is maintained within its theoretical limit.

(d) **TARC**

Finally, the TARC parameter measures the diversity performance in terms of ratio of total reflected power to that of total incident power across the designed MIMO antenna and is evaluated using equation (9) [20]:


$$\begin{array}{c} TARC=\frac{\sqrt{\sum_{k=1}^{N}{\left|b_{k}\right|}^{\text{2}}}}{\sqrt{\sum_{k=1}^{N}{\left|a_{k}\right|}^{\text{2}}}} \end{array}$$ (9)

**Fig 23:**
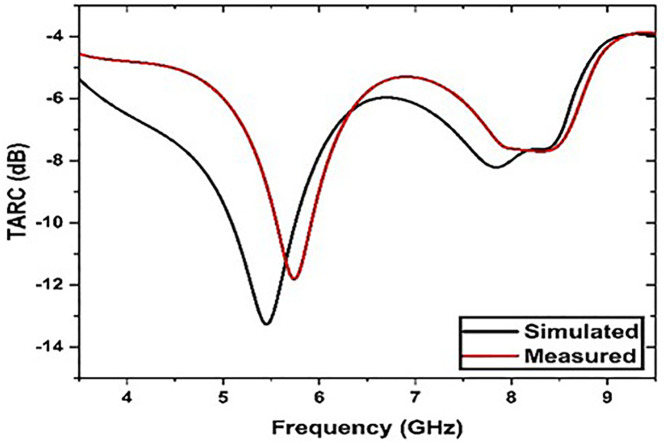
TARC of the proposed design.

Here, a_k_ and b_k_, represents the incident and the reflected wave at the k_th_ port respectively with N denoting the total number of ports. The TARC parameter ideally should be of less than 0 dB value and preferably below −10 dB in the operating region, and must ensure the reflection of 10% of the active input power. The measured TARC (τ) value compared to its simulated value as shown in Fig 23 w.r.t. frequency (GHz). Also, the designed MIMO antenna exhibits TARC < −10 dB at the desired resonant frequencies, indicating efficient power transmission. Thus, the proposed triple band element MIMO antenna depicts optimal TARC performance in the desired frequency bands ensuring minimal reflections.

## 6. Optimization of the anticipated antenna design with machine learning

**Fig 24:**
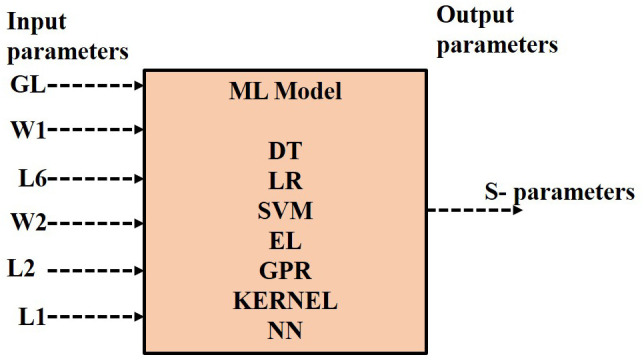
Input and output parameters of the ML algorithms.

**Fig 25:**
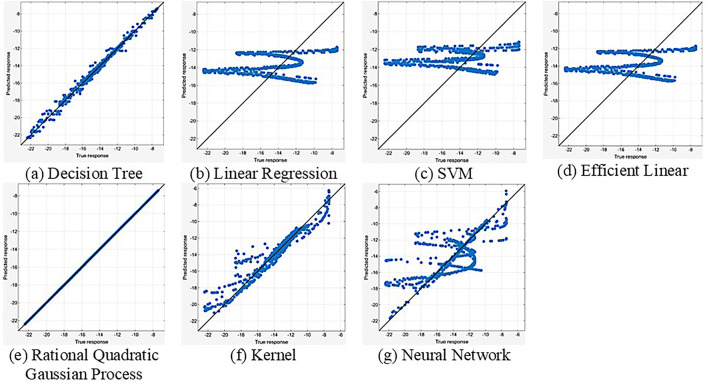
Actual vs. forecast values of various ML algorithms.

ML algorithms are used to envisage S_11_(dB) of the anticipated antenna. Selecting a ML technique helps to reduce simulation time and errors. The performance and the resonating frequencies of the anticipated antenna depends on various dimensional parameters which includes the length of the patch (L1), feed width (W2), length of the ground plane (GL) etc. [21–23]. These variables will act as the input of the ML models as depicted in Fig 24. Each parameter was varied within physically realizable ranges derived from antenna design constraints and resonance requirements, ensuring coverage of the feasible design space. The dataset was generated through systematic parametric variation of the proposed MIMO antenna geometry using full-wave EM simulations in HFSS. Each data sample corresponds to a complete EM simulation. Approximately 400 samples were generated through structured parametric sweeps. The dataset was randomly divided as: 70% Training, 15% Validation, 15% Testing while maintaining uniform distribution across the design space. The Mean Square Error (MSE), R^2^ Score, and Mean Absolute Error (MAE) are given in Table 1. MSE justifies the sensitivity to large prediction errors, MAE details the average prediction deviation and R^2^ quantifies the goodness of fit and explained variance. The predicted vs true responses of all algorithms used is shown in Fig 25, which shows the very similar response in case of linear regression and efficient linear and it can also be confirmed from the values provided in Table 1. But the optimized and the best response can be observed in the Rational Quadratic Gaussian Process.

**Table 1:** Different ML model behaviors.

Model Type	MSE	R^2^ Score	MAE
Tree	0.0753	0.9941	0.177
Linear Regression	11.6077	0.0907	2.7593
SVM	12.3016	0.0364	2.693
Efficient Linear	11.6076	0.0907	2.7593
Gaussian Process	1.94E-05	1	0.0017
Kernel	0.9921	0.9223	0.6259
Neural Network	8.2596	0.353	2.1588

**Fig 26:**
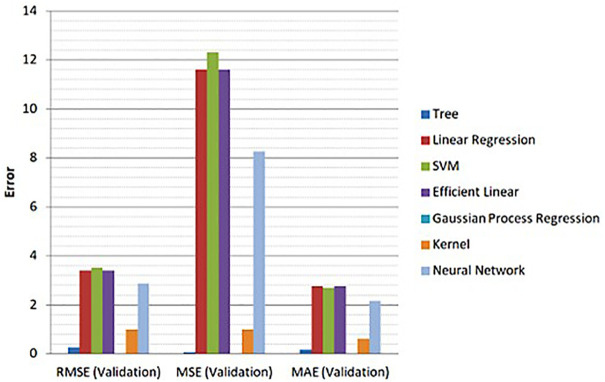
Error Comparison.

**Fig 27:**
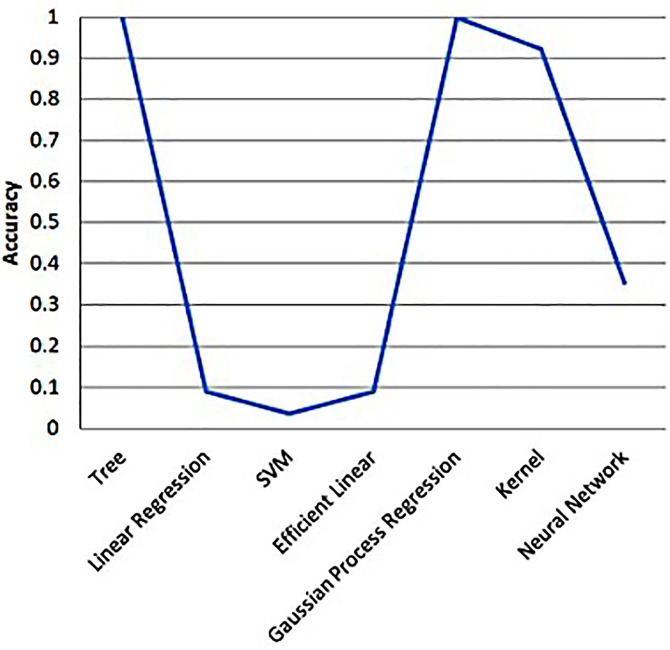
Accuracy comparison.

**Fig 28:**
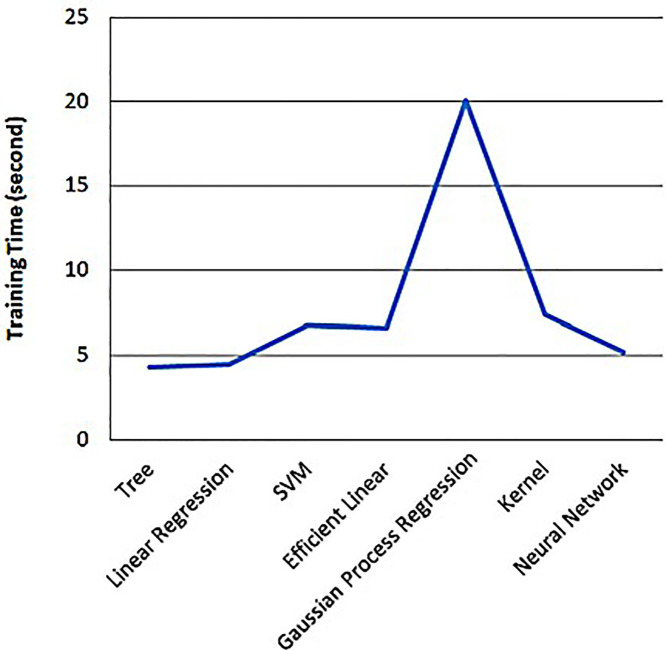
Training Time comparison.

Further, error and accuracy comparison of all algorithms is shown in Fig 26 and 27 and it can be predicted that Gaussian process regression shows the minimum error and maximum accuracy. At last, Training time comparison of 7 all algorithms is shown in Fig 28, which shows the time taken in seconds to train the particular ML model. This ML framework is then used as a substitute optimization tool to learn the relationship between antenna dimensions and S₁₁ performance. Among the tested models, Gaussian Process Regression (GPR) achieved the best accuracy and was used to predict parameter combinations that improve impedance matching and resonance behavior.

**Fig 29:**
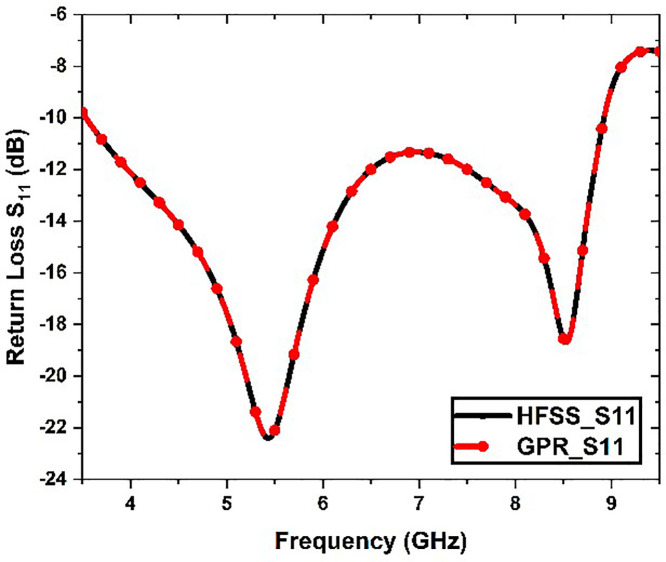
Comparison of best ML algorithms with HFSS.

From the above discussion and analysis, it can be concluded that Gaussian process regression shows the minimum errors and maximum accuracy and there is a close agreement between the actual and predicted values as depicted in Fig 25 and it one of rare phenomenon of such an exact result. Therefore, simulated and predicted S_11_ graphs will exactly match with the frequency variations as depicted in Fig 29. Table 2 presents a comparative performance analysis of various MIMO antenna designs against the proposed design based on key parameters such as number of ports, physical size, impedance bandwidth, electrical size (in terms of wavelength), peak gain, isolation, and ECC. Most referenced designs ([8,14]– [17]) employ 4-port configurations similar to the proposed work, while [11] uses a 2-port system. In terms of size, the proposed antenna (52 × 52 × 1.57 mm³) achieves a compact structure comparable to other designs except [14] but significantly smaller than larger designs like [16] and [17]. The impedance bandwidth of the proposed work (3.5–9.05 GHz) is relatively wide, offering better operational flexibility over the other similar designs which are narrowband or multi-band designs. Its electrical size (0.6 × 0.6 λ₀) indicates a compact form relative to wavelength, aligning well with efficient miniaturization, which is far better than [16] and [17]. The proposed antenna achieves a higher peak gain than most of the reported designs in the literature, with the exception of [8]. However, this gain enhancement is attained at the cost of a relatively larger antenna size. Importantly, the isolation performance of the proposed design (−20 to −40 dB) is superior, indicating excellent port decoupling, while the ECC (<0.05) confirms strong diversity performance and low correlation between antenna elements. Overall, the proposed antenna demonstrates a balanced improvement in compactness, bandwidth, gain, and isolation compared to existing designs.

**Table 2:** Performance comparison of SSFEMIMOA.

Ref	No. of ports	Size (mm^3^)	Impedance BW (GHz)	Size (λ_0_ × λ_0_)	Peak Gain (dB)	Isolation (dB)	ECC	Rad Eff (%)
[8]	4	70 × 70 × 1.575	2.5-50	0.62 × 0.62	8.5	−25	0.013	84%
[11]	2	37 × 56 × 1.6	2.24-2.5 3.6-3.99 4.4-4.6 5.71-5.9	0.27 × 0.4	2	<−10	<0.08	83%
[14]	4	30 × 40 × 1.6	3.2-5.85	0.6 × 0.48	3.5	−20	<0.05	81%
[15]	4	55 × 55 × 1.6	3.34-9.2	0.61 × 0.61	4.3	−20	<0.17	70%
[16]	4	150 × 75 × 6.2	3.4-3.6, 4.8-5.0	1.69 × 0.85	5	−16.5	0.01	85%
[17]	4	160 × 40 × 0.5	4.78-4.82	2.44 × 0.63	5.3	−32	<0.5	N.A.
[24]	2	40 × 49.2 × 1.6	2.61-7.64	0.69 × 0.22	6.67	56	0.05	75-99
[25]	4	178 × 178 × 14	2.76-4.3	1.6 × 1.6	6.2-10.5	−23	0.001	98
This Work	4	52 × 52 × 1.57	3.5-9.05	0.6 × 0.6	6.01	−20 to −40	<0.05	85%

## 7. Conclusion

In this work, a modified SSFEMIMOA has been analyzed and experimentally validated. The proposed antenna achieves a wide impedance bandwidth of 5.5 GHz along with high isolation reaching up to −40 dB. The radiation characteristics demonstrate omnidirectional and bidirectional patterns at 5.67 GHz and 8.49 GHz, respectively. In addition, the antenna maintains a compact form factor compared to similar designs, while providing an acceptable peak gain of approximately 6 dB. The diversity performance parameters, including ECC and radiation efficiency, remain within desirable limits, indicating reliable MIMO operation. Furthermore, the antenna design has been optimized using machine learning techniques, where Gaussian Process Regression demonstrates improved prediction accuracy and reduced error, showing good agreement between simulated and predicted results. It is important to note that the ML framework serves as a preliminary optimization tool to enhance the design process. Overall, the measured and simulated results are in close agreement, confirming the validity of the proposed design. Based on the obtained results, the antenna demonstrates promising performance as a compact wideband MIMO system, while its applicability to specific wireless standards depends on further system-level validation and integration.
